# Introducing a gatekeeping system for amyloid status assessment in mild cognitive impairment

**DOI:** 10.1007/s00259-022-05879-6

**Published:** 2022-07-14

**Authors:** E. Doering, M. C. Hoenig, G. N. Bischof, K. P. Bohn, L. M. Ellingsen, T. van Eimeren, A. Drzezga, for the Alzheimer’s Disease Neuroimaging Initiative

**Affiliations:** 1 German Center for Neurodegenerative Diseases (DZNE), Bonn-Cologne, Germany; 2 University of Cologne, Faculty of Medicine and University Hospital Cologne, Department of Nuclear Medicine, Multimodal Neuroimaging Group, Cologne, Germany; 3 Institute for Neuroscience and Medicine II-Molecular Organization of the Brain, Research Center Juelich, Jülich, Germany; 4 Klinikum Dritter Orden, Department of Radiology and Nuclear Medicine, Munich, Germany; 5 Department of Electrical and Computer Engineering, Johns Hopkins University, Baltimore, MD USA; 6 Department of Electrical and Computer Engineering, University of Iceland, Reykjavik, Iceland; 7 University of Cologne, Faculty of Medicine and University Hospital Cologne, Department of Neurology, Cologne, Germany

**Keywords:** Neurodegeneration, Machine learning

## Abstract

**Background:**

In patients with mild cognitive impairment (MCI), enhanced cerebral amyloid-β plaque burden is a high-risk factor to develop dementia with Alzheimer’s disease (AD). Not all patients have immediate access to the assessment of amyloid status (A-status) via gold standard methods. It may therefore be of interest to find suitable biomarkers to preselect patients benefitting most from additional workup of the A-status. In this study, we propose a machine learning–based gatekeeping system for the prediction of A-status on the grounds of pre-existing information on APOE-genotype ^18^F-FDG PET, age, and sex.

**Methods:**

Three hundred and forty-two MCI patients were used to train different machine learning classifiers to predict A-status majority classes among APOE-ε4 non-carriers (APOE4-nc; majority class: amyloid negative (Aβ-)) and carriers (APOE4-c; majority class: amyloid positive (Aβ +)) from ^18^F-FDG-PET, age, and sex. Classifiers were tested on two different datasets. Finally, frequencies of progression to dementia were compared between gold standard and predicted A-status.

**Results:**

Aβ- in APOE4-nc and Aβ + in APOE4-c were predicted with a precision of 87% and a recall of 79% and 51%, respectively. Predicted A-status and gold standard A-status were at least equally indicative of risk of progression to dementia.

**Conclusion:**

We developed an algorithm allowing approximation of A-status in MCI with good reliability using APOE-genotype, ^18^F-FDG PET, age, and sex information. The algorithm could enable better estimation of individual risk for developing AD based on existing biomarker information, and support efficient selection of patients who would benefit most from further etiological clarification. Further potential utility in clinical routine and clinical trials is discussed.

**Supplementary Information:**

The online version contains supplementary material available at 10.1007/s00259-022-05879-6.

## Introduction

In patients with mild cognitive impairment (MCI), enhanced accumulation of extracellular amyloid-β plaques and pronounced neurodegeneration reliably predict the development of dementia with Alzheimer’s disease (AD) [1]. MCI diagnoses entail that an individual has cognitive complaints and objective impairment in one or more cognitive domains, in the absence of dementia or impairment in functional everyday life [2]. Disease prognoses are of impeccable importance to MCI patients to make provision for the future, as well as to obtain access to treatment or clinical trials, which are usually contingent on a positive amyloid status (A-status) [e.g., 3]. A-status can be assessed via positron emission tomography (PET) imaging or via the analysis of beta-amyloid markers derived from cerebrospinal fluid (CSF). A positive A-status on PET indicates high cerebral amyloid pathology and is a key inclusion criterion for several clinical trials on anti-amyloid medications [4]. Moreover, PET A-status avoids signal fluctuations, which are frequently reported for CSF biomarkers [5], and no contraindications are known for PET, while some exist for lumbar puncture (e.g., anticoagulants), which is necessary for CSF acquisition. Not all patients have access to the assessment of various biomarkers to clarify individual aetiology of cognitive impairment, due to availability (financial or logistic). It may therefore be of interest to find suitable biomarkers to preselect patients benefitting most from additional workup of the A-status.

While the A-status enables some insights into individuals’ risk of developing AD, recommendations for a reliable biomarker-based diagnosis of AD include additional quantification of cerebral tau load (T-status), as well as the extent of neurodegeneration (N-status), thus constituting the A-T-N classification scheme [6]. By themselves, amyloid biomarkers are insufficient in monitoring the progression of AD after symptom onset [7]. N-status can be assessed via brain magnetic resonance imaging (MRI) or brain ^18^F-FDG-PET. Besides its utility in the diagnostic process of AD, ^18^F-FDG-PET has proven utility in disentangling several phenotypes of dementia and cognitive decline [8, 9]. In MCI, amyloid burden is inversely correlated with glucose metabolism in the brain [10]. As compared to MRI, ^18^F-FDG-PET more accurately and more timely captures (even very early) neurodegeneration-related changes in the brain [11, 12]. Finally, ^18^F-FDG-PET is relatively inexpensive/broadly available and expertise with interpretation of scans is prevalent. Thus, recent expert consensus recommends ^18^F-FDG-PET ahead of amyloid imaging or other profound examinations in the diagnostic sequence for patients with suspected AD [7]. Moreover, carriership of the ε4 allele of APOE, which represents the greatest genetic risk for Alzheimer’s disease, can be easily assessed by inexpensive, simple blood screening. Although it is currently not part of the diagnostic recommendation guidelines given its limited utility, it is frequently assessed in research settings and it has been associated with enhanced (susceptibility to) amyloid pathology [, 13–15]. The combined observation of information about cerebral glucose metabolism (N-status) and APOE-genotype (genetic predisposition for AD), as well as demographic factors, such as age and sex, enhance prognostic accuracy of AD in patients with MCI [16]. Such information, available to a relevant proportion of patients, may be used to classify A-status in order to facilitate and stratify the diagnosis procedure of MCI. Potentially, such information could be extracted by state-of-the-art machine learning algorithms. Together, these assumptions formed the motivation for the current study.

Recently, multimodal machine learning studies reported moderately successful classification of A-status from various combinations of known AD risk factors, such as carriership of the APOE-ε4 allele, higher age, female sex, and N-status measures [, 17–21]. Despite the clear advantage of ^18^F-FDG-PET to depict disease-related neurodegenerative changes, no studies exist, which incorporate ^18^F-FDG-PET into a multimodal framework for A-status classification. State-of-the-art approaches usually provide no indication of the utility of predicted A-status as a risk factor for progression to dementia despite moderately high misclassification rates (20–25%). The high misclassification rates further indicate that A-status cannot be extracted for all individuals by means of available data. However, it may be possible to substitute amyloid testing for a sub-group of individuals, and thus to effectively select subjects requiring further testing, i.e., to introduce a *gatekeeping system* for A-status classification. In a gatekeeping system, logical “OR gates” enable to create sub-groups of individuals, for whom classification with a specific label is precise (i.e., there is a high number of correctly classified individuals) and efficient (i.e., the number of correctly identified individuals is high).

The goals of this study are two-fold: First, we implemented a first-of-its-kind multimodal gatekeeping system for A-status classification in MCI patients, which identifies amyloid negative (Aβ-) or amyloid positive (Aβ+) individuals with high precision. To do so, we introduced an “OR gate,” where participants are split into groups of APOE-ε4 non-carriers (APOE4-nc) and APOE-ε4 carriers (APOE4-c), and subsequently trained classifiers to predict either Aβ- or Aβ+, respectively, based on ^18^F-FDG-PET, age, and sex within these groups. Among APOE4-nc and APOE4-c, the predominant A-status, i.e., the *majority class*, is Aβ- and Aβ+, respectively. By exploiting machine learning algorithms’ inherent preference for majority class predictions and simultaneously requiring high precision of such predictions, we expected to achieve high efficiency of the gatekeeping system. Second, we compared the risk of progression to dementia of multimodality-predicted and gold standard A-status to test whether multimodality-predicted A-status has the potential to substitute gold standard amyloid testing for the identified individuals.

## Method

### ADNI data

Baseline ^18^F-FDG-PET scans of 588 MCI patients used in the preparation of this article were obtained from the Alzheimer’s Disease Neuroimaging Initiative (ADNI) database adni.loni.usc.edu. The primary goal of ADNI has been to test whether biological markers and clinical and neuropsychological assessment can be combined to measure the progression of MCI and AD. Inclusion criteria were (1) a diagnosis of MCI in accordance with the recommended diagnostic National Institute on Aging and Alzheimer’s Association guidelines [2], (2) the availability of ^18^F-FDG-PET and amyloid PET biomarkers at baseline, and (3) availability of information on age, sex, and APOE-genotype. We excluded participants (1) who showed contradictory information about A-status across PET and CSF A-status (see section “Amyloid status”) and (2) who showed standard uptake value ratios (SUVr) above three times the interquartile range in ^18^F-FDG-PET scans (see section “18F-FDG-PET acquisition and pre-processing”).

### Internal memory clinic sample for external validation

To test generalization performance of the gatekeeping systems for APOE4-c and APOE4-nc, we tested our results on an external dataset, an *internal memory clinic* (*IMC*) sample*.* The IMC includes 39 MCI patients for whom ^11^C-PiB-PET scans, ^18^F-FDG-PET scans, and APOE-genotype, age, and sex were available. This dataset was collected at the Technical University of Munich. MCI diagnoses were provided on a value of 0.5 on the clinical dementia rating scale and preserved activities of daily living [22, 23]. All participants provided written informed consent and the study protocol was approved by the ethics committee of the Technical University of Munich.

### Amyloid status

In the ADNI sample, MCI patients’ A-status was assessed by amyloid PET (*n* = 543). PET tracers included ^18^F-florbetaben- (FBB; *n* = 63), ^11^C-Pittsburgh compound-B- (PiB; *n* = 13), or ^18^F-florbetapir-PET (AV45; *n* = 467). Amyloid-PET acquisition and pre-processing details for the ADNI data have previously been published [, 24–27]. Briefly, the scans were co-registered to corresponding MRI images in native space and SUVrs were calculated voxel-wise using the whole cerebellum as a reference region. In the IMC sample, A-status was assessed exclusively via PiB-PET. Scans were acquired on a Siemens scanner 40–70 min (3 × 10 min) after injection with an average dose of 370 MBq (10 MCi) and subsequently co-registered and normalized to an MRI template in native space. Again, SUVrs were extracted using the whole cerebellum as a reference region. A global SUVr score was calculated as the average SUVr from frontal, anterior/posterior cingulate, lateral parietal and lateral temporal (FBB and AV45) or frontal, parietal, precuneus, and anterior cingulate regions (PiB, both samples) [, 24–27]. Finally, global A-status for both samples was determined based on recommended cut-off values defined by SUVR_AV45_ > = 1.11, SUVR_PiB_ > = 1.41, SUVR _FBB_ > = 1.08 [24, 25, 28]. For individuals who additionally received lumbar puncture for amyloid assessment, amyloid-beta_1-42_ peptide in CSF with a cut-off of 192 pg/ml was used to validate amyloid status. Consequently, 51 individuals were excluded from the current dataset (14 who were Aβ negative on CSF, but positive on PET, and 37 who were Aβ positive on CSF, but negative on PET).

### Non-imaging variables

APOE-genotype in all participants was determined from blood samples [29]. We distinguished between APOE-ε4 carriers (APOE4-c), who had at least one APOE-ε4 allele, and APOE-ε4 non-carriers (APOE4-nc), who had no APOE-ε4 allele. Age at clinical diagnosis and sex were available for all participants.

### ^18^F-FDG-PET acquisition and pre-processing

All participants in the ADNI sample received an ^18^F-FDG-PET scan with an average dose of 185 MBq (5 MCi). Scans were acquired dynamically 30–60 min (6 × 5 min frames) post-injection. ^18^F-FDG-PET scans were downloaded with minimal pre-processing (“co-registered, averaged”-format) between November 2020 and February 2021. In the IMC sample, ^18^F-FDG-PET scans were acquired dynamically on a Siemens scanner, 30–50 min (1 × 10 min frame, 2 × 5 min frames) after injection of 185 MBq (5 MCi). Subsequently, frames were averaged over all frames. Using the Statistical Parametric Mapping 12 toolbox (SPM12; www.fil.ion.ucl.ac.uk), we aligned all ^18^F-FDG-PET scans to the anterior commissure*/*posterior commissure*.* Scans were subsequently co-registered and spatially normalized to an MRI template in native space and SUVr images were generated, using the pons as a reference region [30]. Finally, mean regional SUVrs of 90 (non-cerebellar) cortical and subcortical brain regions were extracted using the automated anatomical labeling (AAL) atlas [31]. By means of an outlier analysis, subjects, that were outside of three times the interquartile range of single regions’ SUVRs, were excluded from our sample (*n* = 2). This resulted in the final number of 490 participants.

### Final samples

To train the classifiers, participants from the ADNI and IMC sample were first grouped by APOE-ε4 carriership (APOE4-nc and APOE4-c). Subsequently, stratified splits were created from the ADNI sample, where 70% of the ADNI data were used as a training set, while the remaining 30% and the IMC sample constituted the test sets. Stratified splitting allows to maintain the overall proportion of both classes (Aβ- and Aβ+) in the train set and test set. To compare rates of progression to dementia in the last step of our analyses, only participants from the ADNI test set were considered, who received at least two follow-up diagnoses, at least 6 months apart (*n* = 118).

### Pipeline architectures

This section describes the machine learning pipelines. Briefly, after grouping into APOE4-nc and APOE4-c, we first scaled (section “Scaling”), and then upsampled the data (section “Upsampling”), and finally trained and tested the classifiers. A schematic illustration of the machine learning pipeline is available in the Supplementary Material (Fig. S1).

#### Scaling

Feature scaling is a critical part of pre-processing the input for machine learning classifiers. Data is either normalized; i.e., values are scaled to fall between a pre-defined range, or standardized; i.e., values are scaled to have zero mean and a variance of 1. Here, we standardized age and ^18^F-FDG-PET data (region-wise) and applied the estimated transformation parameters to the test sets. Sex (male = 0; female = 1) was coded in a binary manner.

#### Upsampling

Upsampling refers to the process of randomly sampling data for duplication in the minority class with replacement. Due to the imbalanced nature of the sub-samples, we upsampled the under-represented (i.e., *minority*) class of the train sets (Aβ+ in the APOE4-nc sample; Aβ- in APOE4-c sample) to match the number of participants from the respective majority class. This resulted in training set sizes of *n* = 268 (APOE4-nc) and *n* = 334 (APOE4-c).

#### Classifier training

Previous research has mostly used logistic regression classifiers, a rather simple machine learning classifier, for the classification of A-status in MCI patients. Since there is no existing rationale for complexity of the current task, we tested a variety of simple and more complex classifiers. The same set of six machine learning classifiers (K-nearest neighbours (KNN), support vector machine (SVM), Gaussian process classifier (GPC), a feed-forward deep neural network (DNN), random forest classifier (RFC), and logistic regression (LR)) and the same set of possible hyperparameter configurations per classifier (see Supplementary Table S1) were used for both the APOE4-nc and APOE4-c groups. Hyperparameters are parameters that control the learning process. Here, the optimal combination of hyperparameters for each classifier was determined via ten-fold cross-validated grid search, yielding six *transition models* (one for each classifier). During cross-validation, stratified splits were used to maintain the overall distribution of Aβ-/Aβ + in each fold. A configuration of hyperparameters was considered “optimal,” if it yielded the highest average *F*_1/10_-score across validation folds as compared to all other combinations of hyperparameters in the same classifier. The *F*_1/10_-score is a variant of the *F*_*β*_-score, where *β* refers to the relative contribution of recall (proportion of relevant instances identified) over precision (proportion of correctly classified relevant instances) to the metric (Eq. 1). With *β* set to 1/10, transition models are chosen, which prioritize precision over recall by a factor of 10, thereby enabling a strong focus of correctness of classifications.

1 $${F}_{\beta }=\left(1+ {\beta }^{2}\right)* \frac{\mathrm{Precision}*\mathrm{Recall}}{{(\beta }^{2}*\mathrm{Precision})+\mathrm{Recall}}$$

Finally, the transition model with the best validation score (rounded to full percent) was chosen as the *final model*, and generalizability to the test sets was evaluated on the ADNI and IMC test set, separately. In case several transition models achieved the best validation score, the model with the highest performance on the ADNI test set was chosen and subsequently evaluated on both test sets. All classifiers were implemented using scikit-learn in Python 3.8 on a 64-bit Linux machine with 18 CPU cores and 2 threads per kernel.

To provide an estimate of model overfitting on the majority class, we additionally tested the final model’s performance on randomly downsampled balanced subsets of the ADNI sample (*n*_APOE4-nc_ = 66; *n*_APOE4-c_ = 28), where the number of majority class individuals was reduced to match the minority class. To allow for a reliable estimate, we report average model performance on *n*^2^ randomly downsampled subsets.

### Feature importance

In order to examine the biological plausibility of the models, we calculated each feature’s *permutation importance*. Using permutation importance, the importance of single features is assessed by randomly shuffling an input feature, before providing the complete feature set to a trained classifier. When important features are permuted, model performance will decrease. Inverting the margin of performance decrease yields the permutation importance (*δ*), where higher values correspond to more important features. Here, we permuted each feature 1000 times and calculated the average *δ* on the (ADNI) test set [32].

Due to the novelty of this approach, no trivial baseline could be established for comparison. Therefore, to validate our models, we performed an ablation study, wherein all classifiers were trained either on the whole training set (CL) or grouped by APOE4 carriership, i.e., following the gatekeeping approach (GK). During training, models maximized balanced accuracy (BA; see (2))

2 $$\mathrm{Balanced}\;\mathrm{Accuracy}=\frac{\mathrm{Sensitivity}+\mathrm{Specificity}}2$$

or the *F*_1/10_-score (F1/10). Furthermore, we also assessed classification performance on the full set of features versus on reduced subsets. In the latter, either ^18^F-FDG-PET signal, APOE-ε4 carriership, age, or sex was completely removed from the input (e.g., age was removed in GK-age), or only a specified percentage (10% or 50%) of “ad-hoc most relevant” features were provided (e.g., only the 10% of features were provided in GK-sub10). Ad-hoc feature relevance was determined with the mutual information criterion, which quantifies the dependency between an input feature and outcome (here: A-status) as the reduction of entropy in the outcome given a specific input feature. The most entropy-reducing input features were considered “most relevant”.

### Comparison of multimodality-predicted and gold standard A-status in dementia risk assessment

By comparing the risk of progression to dementia between multimodality-predicted and gold standard A-status, we aimed to test whether predicted A-status is comparably indicative of risk of progression to dementia as gold standard A-status. To do so, we considered subsets of APOE4-nc and APOE4-c participants from the ADNI test set, who had at least two follow-up diagnoses after baseline. Subsequently, we compared the proportion of individuals who received a diagnosis of dementia during follow-up between gold standard and multimodality-predicted Aβ- in APOE4-nc and between gold standard and multimodality-predicted Aβ+ in APOE4-c by means of the *χ*^2^ test with a significance level of 0.05. Dementia at follow-up was defined by the ADNI standard, which included MMSE scores between 20 and 26 (inclusive), a CDR of 0.5 or 1.0, and individuals had to meet the NINCDS/ADRDA criteria for probable AD [33]. Progression to dementia was further sub-divided into three categories: (1) Probable AD (participants who were amyloid positive as determined by AV45 PET at or before time point of diagnosis), (2) non-AD dementia (participants who were amyloid negative as determined by AV45 PET at time point of diagnosis or later), and (3) possible AD (participants where AV45 PET was not available at time point of dementia diagnosis).

## Results

### Participant characteristics

Scans of 529 MCI patients (490 subjects from the ADNI and 39 subjects from the IMC) were split into two groups: APOE4-nc (*n*_APOE4-nc_ = 249) and APOE4-c (*n*_APOE4-c_ = 241). Classifiers were cross-validated on 70% of the ADNI data, and tested on the remaining 30%, as well as the IMC sample. Table 1 and Supplementary Tables 1 and 2 show the clinical characteristics of the training and both test sets. All subjects were on average (SD) 71.6 (7.4) years old and 223 (46%) subjects were female. Two hundred eighty-one participants (57%) were Aβ+. There was no significant difference of sex between Aβ+ and Aβ- participants. The time interval between the ^18^F-FDG-PET and amyloid assessment was on average 13 days (SD = 84 days).

**Table 1:** Training sample demographics

	APOE4-nc sample		APOE4-c sample	
	Aβ-	Aβ+	Aβ-	Aβ+
n	117	57	29	139
Mean age [years (SD)]	70.6 (7.65)	74.5 (7.25)	67.1 (8.08)	72.4 (6.59)
Sex (%Female)	46%	42%	45%	42%
Ethnicity (%White)	90%	96%	86%	94%
Global AV45 [SUVR (SD)]	1.00 (0.05)	1.37 (0.17)	1.00 (0.06)	1.40 (0.16)
MMSE (SD)	28 (1.64)	28 (1.70)	29 (1.50)	27 (1.89)
CDR sum boxes (SD)	1.31 (0.84)	1.61 (0.86)	1.16 (0.67)	1.78 (1.03)

*Global AV45*, mean global SUVR with most frequently used AV45 tracer; *CSF*, mean amyloid-beta_1–42_ peptide in CSF; *MMSE*, Mini-Mental State Exam

### APOE-ε4-dependent gatekeeping of amyloid status

In APOE4-nc, KNN outperformed all other models in the classification of Aβ- during ten-fold cross-validation (see Supplementary Fig. S2), yielding a mean *F*_1/10_-score of 94%. On the ADNI test set, an F_1/10_-score of 87% was reached, which was constituted by a precision of 86%, and a recall of 51%, thus demonstrating high performance on unseen data. On the IMC, an *F*_1/10_-score of 71% was reached, composed of a precision of 71% and a recall of 50%, indicating that inter-dataset generalizability was limited. To deliver an estimate of model overfitting, we calculated average *F*_1/10_-scores on randomly downsampled, balanced subsets of the ADNI and IMC test sets, in which the number of Aβ- individuals was reduced to match the number of Aβ+ individuals. A high average *F*_1/10_-score of 75% was maintained on the balanced ADNI test set (precision: 75%; recall: 51%), thus proving to be relatively stable against data imbalance.

**Fig. 1:**
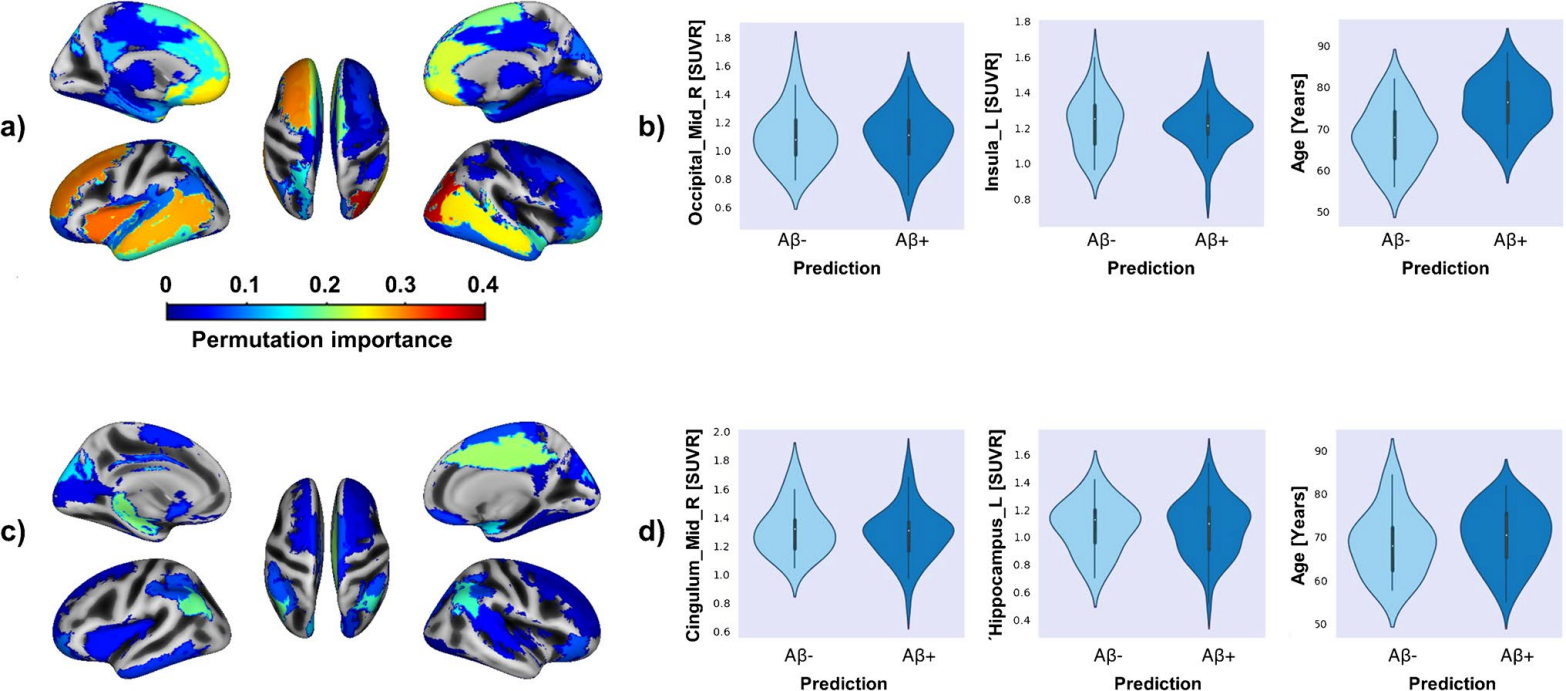
Features relevant for the classification of Aβ- in APOE4-nc (top row) and Aβ+ in APOE4-c (bottom row). Thresholded at 0.001 for visibility. (**a**) Several left-hemispheric, especially frontal and temporal brain regions were highly important for the classification of Aβ- in APOE4-nc. The two most important single brain regions and age (the most important non-imaging feature) are displayed in (**b**), delineating that while Aβ- individuals were younger, SUVR distribution in single relevant brain regions was not different between predicted Aβ- and Aβ + . (**c**) AD-typical brain regions were relevant for the classification of Aβ+ in APOE4-c. Violin plots of SUVR in the two most relevant brain regions and age (irrelevant to the classification task, depicted for comparison to APOE4-nc) are shown in (**d**). Inflated brain representation created with cat12

Permutation importance analyses revealed that high ^18^F-FDG-PET signal in the right middle occipital lobe (*δ* = 0.037), as well as several frontal (left insula, left middle frontal lobe, left superior frontal lobe, left and right medial orbitofrontal lobe, left and right rectus), and temporal regions (left and right middle temporal lobe and temporal pole) was most indicative of Aβ- in APOE4-nc (Fig. 1a). Across test subjects, signal distribution in Aβ- and Aβ+ was not different, thus indicating that metabolism in networks of brain regions, rather than individual brain regions, caused the algorithm’s high performance. Lower age (*δ* = 0.023) was moderately indicative of Aβ- (Fig. 1b), while sex (*δ* = 0.007) had low impact on the classification outcome.

In APOE4-c, an SVC outperformed all other models in the classification of Aβ+ in the ten-fold cross-validation stage, while a total of three classifiers reached close to 100% during cross-validation (see Supplementary Fig. S2). On the ADNI test set, an *F*_1/10_-score of 87% was reached, which was constituted by a precision of 87%, and a recall of 79%, thus indicating high performance on unseen data. On the IMC dataset, *F*_1/10_, precision, and recall scores of 91%, 91%, and 95% were reached. However, analyses on the balanced subsets showed that the majority class was predicted more frequently, and the average *F*_1/10_-score was only 57% (precision: 57%; recall: 100%). Optimal hyperparameter configurations for the classification of A-status in APOE4-nc and APOE4-c can be found in the Supplementary Materials.

Permutation importance analyses showed that ^18^F-FDG-PET signal in the right middle cingulate gyrus, bilateral angular gyrus, and subcortical regions (left hippocampus and right amygdala) pre-dominantly influenced classification of Aβ+ in APOE4-c. Again, no difference of SUVR distribution was detected across subjects in these regions (Fig. 1c). Age and sex did not influence classification outcome.

Finally, we performed an ablation study, wherein we investigated the importance of our input features, as well as the gatekeeping approach. Table 2 demonstrates that the proposed gatekeeping algorithm with the complete feature sets yielded more precise classifications than conventional classification, or gatekeeping algorithms trained on only a subset of data. Ablation of all ^18^F-FDG-PET features in both groups resulted in lower precision, but the highest recall across all feature subsets, suggesting that without ^18^F-FDG-PET features, distribution-based affinity for majority class prediction was stronger, thereby proving the importance of consideration of ^18^F-FDG-PET signal, especially in APOE4-nc. The overall small differences in *F*_1/10_-score observed in ablating features for the APOE4-c gatekeeping algorithms suggest that the input features did not significantly improve a priori probabilities and is in line with the severe overfitting observed when testing the classifier on balanced subsets.

**Table 2:** Ablation study demonstrating the gatekeeping system’s high performance on both Aβ + and Aβ- classification

	Aβ+			Aβ-		
	*F*_1/10_	Precision	Recall	*F*_1__/10_	Precision	Recall
CL^ba^	73%	73%	67%	68%	58%	54%
CL^ba^-APOE	64%	64%	67%	53%	53%	38%
CL^F1/10+^	73%	73%	64%			
GK ^F1/10+^	**87%**	**87%**	79%			
GK-FDG ^F1/10+^	85%	85%	**85%**			
GK-age^F1/10+^	87%	87%	77%			
GK-sex^F1/10+^	83%	83%	82%			
GK-sub10 ^F1/10+^	86%	86%	79%			
GK-sub50 ^F1/10+^	83%	83%	79%			
CL^F1/10−^				63%	64%	57%
GK^F1/10−^				**86%**	**87%**	51%
GK-FDG ^F1/10−^				76%	76%	**73%**
GK-age^F1/10−^				84%	84%	53%
GK-sex^F1/10−^				84%	84%	53%
GK-sub10 ^F1/10−^				82%	82%	63%
GK-sub50 ^F1/10−^				78%	78%	71%

*CL*, classification without gatekeeper, including APOE-genotype unless marked by “-APOE”; *GK*, classification with gatekeeper; *CL-APOE*, classifier trained without APOE carriership; *GK-FDG*, gatekeeper trained without.^18^F-FDG-PET; *GK-age*, gatekeeper trained without age, *GK-sex*, gatekeeper trained without sex; *GK-sub10/GK-sub50*, gatekeeper trained on reduced feature set (10%/50% of features with highest mutual information). *F1/10* + , trained on F_1/10_-score of Aβ+ ; *F1/10-*, trained on F1/10-score of A﻿β-; *ba*, trained on balanced accuracy. Bold font shows column-wise highest value

### Comparison of multimodality-predicted and gold standard A-status in dementia risk assessment

In the second part of our analyses, we investigated whether multimodality-predicted A-status provided risk estimates for progression to dementia comparable to the risk indicated by gold standard A-status. In the previous step, classifiers were chosen that maximized the *F*_1/10_-score of the majority class (Aβ- in APOE4-nc and Aβ + in APOE4-c); therefore, no consideration was given to predicted and gold standard minority classes. At least two follow-up scans were available for a subset of 60 test subjects in the APOE4-nc (80%) and 58 individuals from the APOE4-c group (79%). The average (SD) follow-up time was 56 (30) months for APOE4-nc and 49 (30) months for APOE4-c.

In the APOE4-nc group, five of 41 Aβ- individuals (12%) were diagnosed with dementia in following years. In comparison, zero out of 23 predicted Aβ- individuals (0%) progressed to dementia. Of the five gold standard Aβ- individuals who converted to dementia, four were possible AD patients and one had non-AD dementia. Thus, predicted amyloid negativity potentially depicts long-term stability against cognitive decline of unresolved aetiology in MCI patients as good, or even slightly better than true amyloid negativity.

Among APOE4-c, 21 out of 49 Aβ + individuals (43%) received a diagnosis of dementia in the following years. In comparison, 17 of 43 predicted Aβ + individuals (40%) progressed to dementia. All of these individuals were also Aβ+ as assessed by gold standard methods, thus constituting no difference between progression to dementia between gold standard and predicted A-status. None of the gold standard or predicted Aβ+ individuals were Aβ- at follow-up; thus, all dementia diagnoses were of AD type. Therefore, risk of dementia and AD was equal as assessed by gold standard and predicted Aβ + in APOE4-c.

## Discussion

**Fig. 2:**
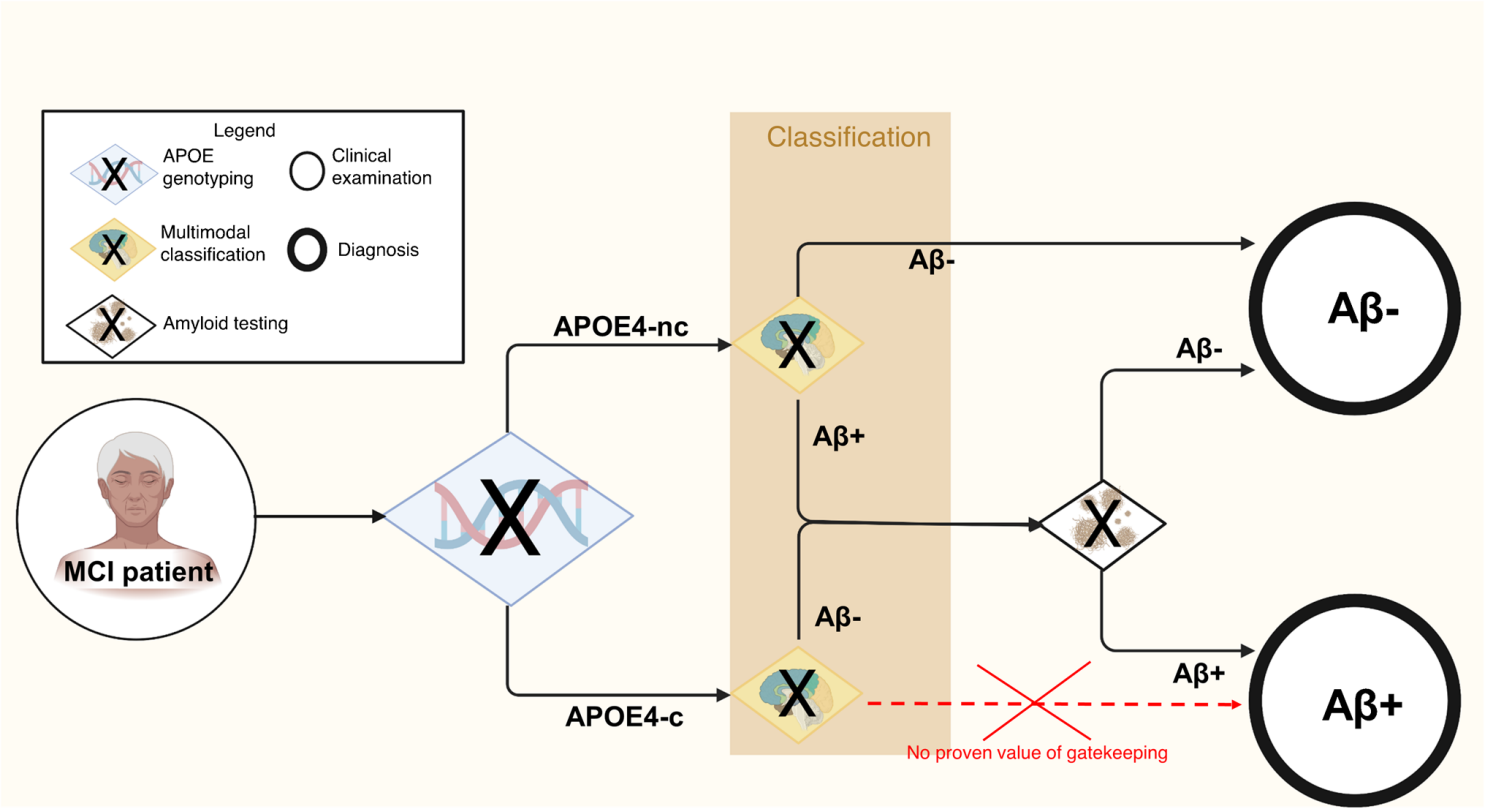
Suggested gatekeeping system with “OR” (X) gates enabling reduction in the overall need for amyloid testing in MCI patients. After screening for APOE carriership, individuals’.^18^F-FDG-PET scans as well as age and sex information provided to the respective machine learning classifier. In case of an Aβ- prediction for APOE4-nc, explicit amyloid testing can be spared. Figure created with BioRender

In this study, we implemented and validated a first-of-its-kind APOE-dependent gatekeeping system, by means of which A-status can be determined for a subgroup of MCI patients from ^18^F-FDG-PET, age, and sex with high precision. Notably, we also demonstrated that predicted A-status is at least equally indicative of risk of progression to dementia as gold standard A-status, thereby highlighting its clinical and scientific utility. For APOE4-c, poor performance of the classification algorithm on the balanced test sets indicated limited benefit of the gatekeeping approach in this group. Based on these results, we suggest a pipeline that directs further action in the assessment of A-status in MCI depicted in Fig. 2. Whereas all APOE4-c recommended an amyloid PET or CSF assessment of amyloid, APOE4-nc only receive amyloid testing based on the specific results of the classification procedure.

Following the A-T-N classification scheme proposed by Jack and colleagues, to be considered as having underlying AD pathology, MCI patients must have a positive A-status [6]. Consequently, key eligibility criteria for the prescription of newly emerging anti-amyloid drugs, such as aducanumab (Aduhelm™), as well as clinical trials thereof, include A-status assessment [e.g., 3]. However, observation of a positive T- or N-status aids in confirming an AD diagnosis and in monitoring cognitive decline [6, 34]. For clinical trials, it has been suggested that the additional requirement of a positive N-status enables to considerably reduce required sample sizes by identifying a patient population that is most likely to rapidly convert to AD [35]. The current gatekeeping methodology could simplify the establishment of complete A-T-N classifications in clinical practice and trials through the possibility to cost-efficiently extract some individuals’ A-status from available data, and thus contribute to the exclusion of Aβ- individuals from trials without added risk, cost, and time. Given the strong association of T- and N-markers, it seems likely that the classification of T-status is possible from markers of neurodegeneration, which could be investigated by future research.

In line with previous literature, we found that among APOE4-nc, high brain metabolism in several left hemispheric frontotemporal brain structures was associated with an Aβ- prediction and consequently reduced probability of progressing to AD. These brain regions are known to be progressively affected in the course of AD [36]. Furthermore, left-dominant pathology and metabolic vulnerability in early AD have repeatedly been demonstrated [, 37–39]. Given the overall high performance of Aβ- classification in APOE4-nc, the lack of a clear trend in SUVR distribution in Aβ- vs Aβ + predictions likely points towards the significance of left-dominant metabolism changes in brain networks, rather than single brain regions [40, 41]. Classification of amyloid status from SUVR covariance patterns of tau pathology has previously been demonstrated [42] and presents a promising avenue for future research.

The APOE4-c-based classifiers proved not to be better than chance in the balanced test sets, and ablating even all ^18^F-FDG-PET features resulted in only a small decrease in performance. Importantly, this result strongly underlines the need to understand and question the complex behavior of machine learning classifiers. The observation that the multimodal classification of Aβ+ showed no clinical benefit for APOE4-c likely results from the very high a priori probability of APOE4-c participants in the current study to be Aβ+ , and the fact that amyloid deposition naturally increases with age [43]. While the distinction of amyloid negative and positive individuals is critical for clinical diagnoses and patient selection as described earlier, biologically, amyloid plaque deposition is a continuous process. The current gold standard for an A-status cut-off value is solely based on amyloid measures (PET and/or CSF). However, it was shown that APOE4-nc and APOE4-c display differential susceptibility to amyloid pathology [15] and a recent study found that an amyloid positivity threshold applied as a function of APOE-ε4 carriership best distinguished Aβ+ from Aβ- individuals, with a higher threshold being applied to APOE4-c than APOE4-nc [44]. If APOE-dependent cut-off values are validated in further studies, they might also improve predictability of Aβ+ among APOE4-c with a gatekeeping methodology.

Some limitations of our study should be acknowledged. First, classification performance of Aβ- in APOE4-nc in the IMC was only 71%. Post hoc analyses revealed that test set classification of Aβ- in APOE4-nc who received a PIB-PET in the ADNI sample was 100% precise. However, due to the very low number of participants in the assessed ADNI test sample measured with this tracer (*n* = 3), a reliable estimate of the unique precision in predicting PIB-PET positivity cannot be assessed, and it still appears possible that the drop in performance may have been caused by the low representation of gold standard A-status derived from this specific tracer in the ADNI (*n*_train_= 7). Alternatively, we speculate that this drop in performance may be attributed to the difference in variability of cognitive impairment in MCI populations. In our sample, IMC APOE4-nc showed marginally higher ratings on the CDR sum of boxes (*t*(263) = 1.95, *p* = 0.05) and thus represented an overall more cognitively impaired population, with potentially stronger neurodegenerative patterns present. Another limitation is that in order to minimize efforts to acquire data for amyloid status classification in clinical practice, we refrained from using several imaging modalities in this work. Through the lack of MRI information for the current method, we cannot exclude the possibility that a positive A-status also reflected vascular amyloid deposition, rather than pure cerebral AD pathology. Another limitation is that ADNI data is not representative of the general population, as Caucasian individuals are highly over-represented [45]. For the IMC sample, no information on ethnic background was collected. Epidemiological studies have revealed that there are profound differences in APOE-ε4 prevalence among AD patients, depending on their geographical background, with higher prevalence in Northern/Central Europe and Australia and lower prevalence in Southern Europe and Asia [46]. Other studies confirm location-dependent genotype differences of APOE [47], thus pointing towards AD disease heterogeneity. Given the population bias in our sample, extrapolation to the general population should be done with caution and further studies with currently under-represented groups must be done to validate these findings. We also acknowledge that research building medical gatekeeping systems with machine learning is an uncharted territory. By including conventional classification into the ablation study, we could show that gatekeeping systems provide a significant advantage to the assessment of A-status compared to classification approaches. Yet, future studies must confirm the clinical applicability of the gatekeeping approach, *F*_1/10_-score as the optimal metric, as well as the choice of ^18^F-FDG-PET, age, and sex as optimal input features. It will be especially interesting to assess the gatekeeping methodology using MRI or plasma biomarkers instead of ^18^F-FDG-PET, which have higher availability and lower cost compared to ^18^F-FDG-PET. Recent work on plasma biomarkers has shown that they are associated with gold standard measures of amyloid status [8, 9] and that plasma amyloid biomarkers detect very early abnormal amyloid levels [10]. Thus, plasma biomarkers may represent a well-suited predictor of PET A-status in future machine learning classification approaches. PET A-status is required to start anti-amyloid therapies or clinical trials, as it represents the actual distribution of the therapeutic target (amyloid plaques) in the brain. Usage of plasma or other, fluid biomarkers per se for the stratification of patients is currently not recommended, as this might result in the inclusion of patients before sufficiently abnormal amyloid plaque deposition levels have been reached in the brain. It should be noted, however, that using plasma biomarkers for classification would reduce the amount of information fed to the classifier, which is especially relevant, when considering that these biomarkers show fluctuations, even during the day [6]. Finally, with an ^18^F-FDG-PET scan as the basis of the classification procedure for PET A-status, not only AD, but other potential sources of MCI can be investigated. Nevertheless, implementation of a multivariate gatekeeping approach using several blood biomarkers, e.g., measures of amyloid, neurofilament light, and tau, in combination with available information (clinical/demographic variables) will be an interesting topic for future research.

In conclusion, we developed a first-of-its-kind gatekeeping methodology for the approximation of A-status in MCI based on ^18^F-FDG-PET, age, and sex for APOE4-nc and APOE4-c. The gatekeeping system not only provided highly precise predictions for APOE4-nc, but predicted Aβ- showed similar (possibly even better) risk for progression to dementia as Aβ- assessed with gold standard methods. In the future, the implementation of gatekeeping methodologies could enable better estimation of individual risk for developing AD based on existing biomarker information. In addition, it could support more efficient selection of patients who would benefit from further etiological clarification using additional diagnostic tests.

## Supplementary Information

Below is the link to the electronic supplementary material.

Supplementary file1 (PNG 1441 KB)

Supplementary file2 (PNG 242 KB)

Supplementary file3 (DOCX 544 KB)

## Data Availability

The data that support the findings of this study are available upon request to the corresponding author. The code used for this project is publicly available on the GitHub page of the corresponding author.
