# Supplementary figures and images for: Introducing a gatekeeping system for amyloid status assessment in mild cognitive impairment
Source: Eur J Nucl Med Mol Imaging. 2022 Jul 14;49(13):4478–89. doi: 10.1007/s00259-022-05879-6 (PMC9605923; doi:10.1007/s00259-022-05879-6)

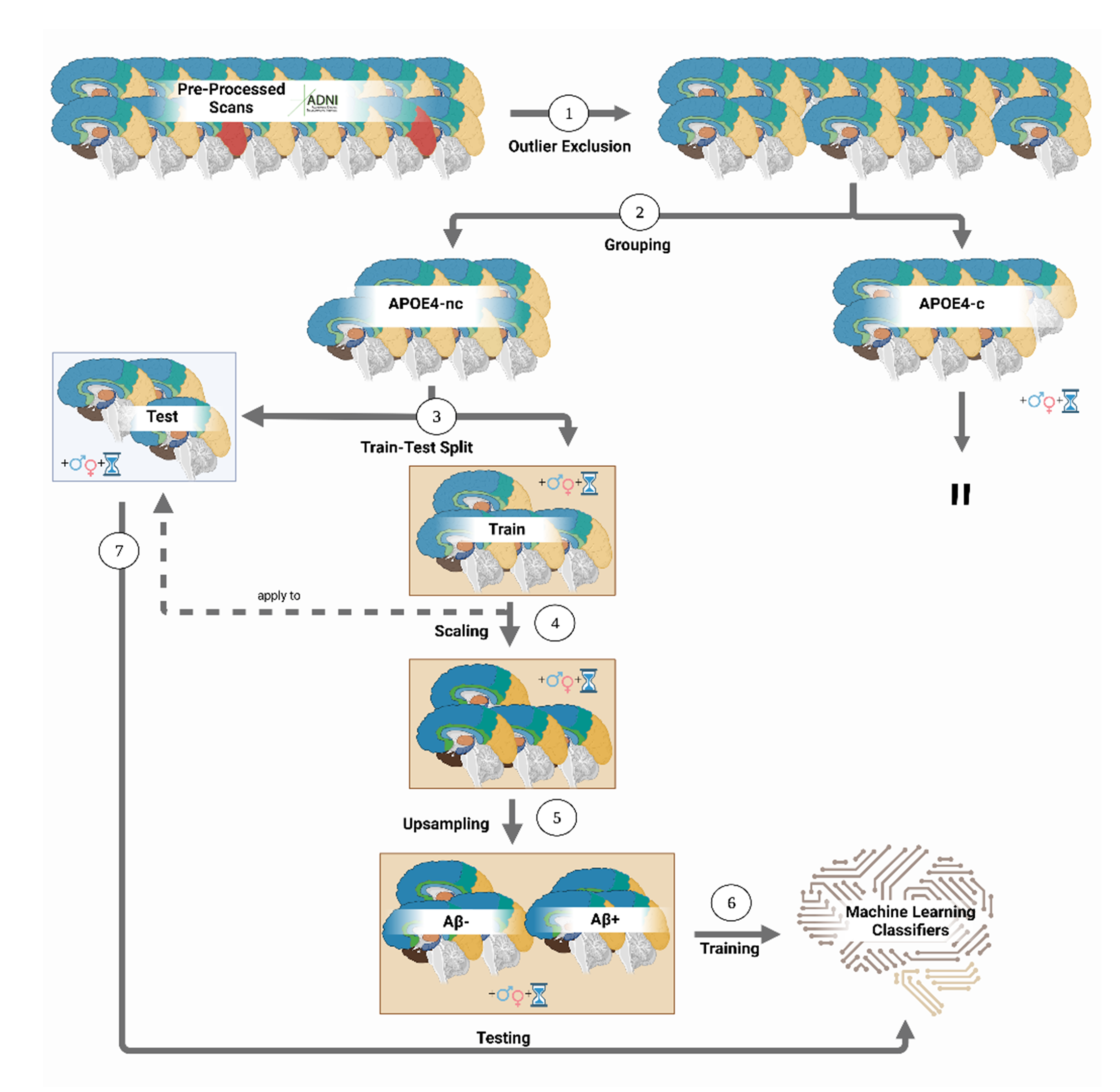

Supplement: Supplementary file 1 — Supplementary file1 (PNG 1441 KB) [file 259_2022_5879_MOESM1_ESM.png]

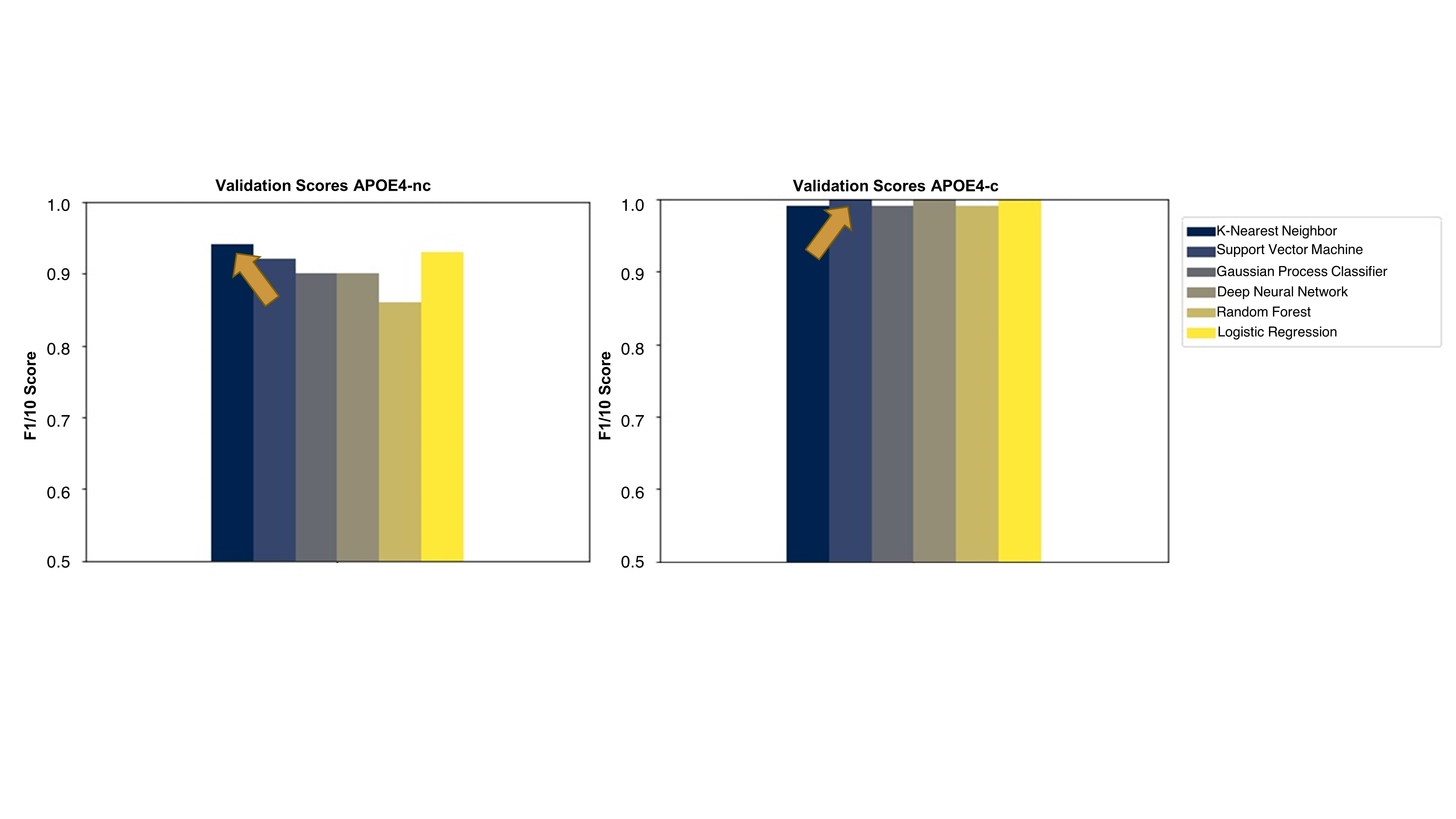

Supplement: Supplementary file 2 — Supplementary file2 (PNG 242 KB) [file 259_2022_5879_MOESM2_ESM.png]
