# Supplementary material for: Introducing a gatekeeping system for amyloid status assessment in mild cognitive impairment
Source: Eur J Nucl Med Mol Imaging. 2022 Jul 14;49(13):4478–89. doi: 10.1007/s00259-022-05879-6 (PMC9605923; doi:10.1007/s00259-022-05879-6)
Supplement: Supplementary file 3 — Supplementary file3 (DOCX 544 KB) [file 259_2022_5879_MOESM3_ESM.docx]

**Supplementary Materials to “Introducing a Gatekeeping System for A-status Assessment in Mild Cognitive Impairment”**

***Table S1****. ADNI Test sample demographics*

|  | ADNI  APOE4-nc | | ADNI  APOE4-c | | |
| --- | --- | --- | --- | --- | --- |
|  | Aβ- | Aβ+ | | Aβ- | Aβ+ |
| n | 51 | 24 | | 12 | 61 |
| Mean age [years] (SD) | 71.8 (7.77) | 75.7 (6.49) | | 64.6 (6.90) | 71.0 (6.37) |
| Sex (%Female) | 43% | 38% | | 58% | 57% |
| Ethnicity (%White) | 96% | 100% | | 92% | 93% |
| Global AV45 (SD) | 1.01 (0.05) | 1.38 (0.18) | | 1.00 (0.05) | 1.41 (0.18) |
| MMSE (SD) | 28 (1.71) | 28 (1.55) | | 29 (1.15) | 28 (1.86) |
| CDR Sum Boxes (SD) | 1.35 (0.76) | 1.19 (1.25) | | 1.42 (0.85) | 1.45 (0.79) |

***Table S2.*** *IMC* *Test sample demographics*

|  | IMC sample  APOE4-nc | | IMC sample  APOE4-c | |
| --- | --- | --- | --- | --- |
|  | Aβ- | Aβ+ | Aβ- | Aβ+ |
| n | 10 | 6 | 3 | 20 |
| Mean age [years] (SD) | 70.5 (5.0) | 69.3 (11.0) | 66.0 (13.0) | 72.35 (6.0) |
| Sex (F/M) | 3/7 | 3/3 | 3/0 | 8/12 |
| CDR Sum Boxes (SD) | 1.75 (0.72) | 1.92 (0.58) | 2.50 (0.87) | 2.23 (0.88) |

| ***Table S3.*** Hyperparameters of the Classifiers | |
| --- | --- |
| **K-Nearest Neighbors** | |
| n_neighbors | list(range(2,16,1)+[20, 25, 30, 35, 40, 45, 50] |
| weights | [‘uniform’, ‘distance’] |
| distance | [‘minkowski’, ‘manhattan’] |
| **Support Vector Machine** | |
| kernel | [‘linear’, ‘rbf’] |
| C | [0.001, 0.01, 1, 10, 100, 200, 300] |
| distance | [‘scale’, ‘auto’] |
| **Gaussian Process Classifier** | |
| Only default hyperparameters were considered as no categorical hyperparameter configurations were available. | |
| **Feed-Forward Deep Neural Network (MLP Classifier)** | |
| hidden_layer_sizes | All 34 possible combinations with replacement with [4, 8, 16] nodes per layer in [1,2,3,4] layers |
| width | [2, 4, 8, 16] |
| alpha | numpy.logspace(-5,3,5) |
| solver | [‘sgd’, ‘adam’] |
| batch_size | [4, 8] |
| activation | [‘tanh’, ‘relu’] |
| **Random Forest** | |
| **n_estimators** | [5, 25, 50, 75, 100] |
| **max_depth** | numpy.linspace(3,10,8) |
| **min_samples_split** | [0.2, 0.3, 0.4, 0.5, 0.6, 0.7, 0.8] |
| **criterion** | [‘gini’, ‘entropy’] |
| **Logistic Regression** |  |
| **penalty** | [‘l1’, ‘l2’, ‘elasticnet’] |
| **solver** | [‘newton-cg’, ‘lbfgs’, ‘liblinear’] |
| The following classifier and hyperparameters were determined optimal during grid search in the APOE4-nc group: Classifier = K-Nearest Neighbors, n_neighbors = 40, weights = distance, weights = manhattan. Optimal classification performance in APOE4-c was achieved with the Support Vector Machine and the following hyperparameters: C = 10, kernel = linear. | |


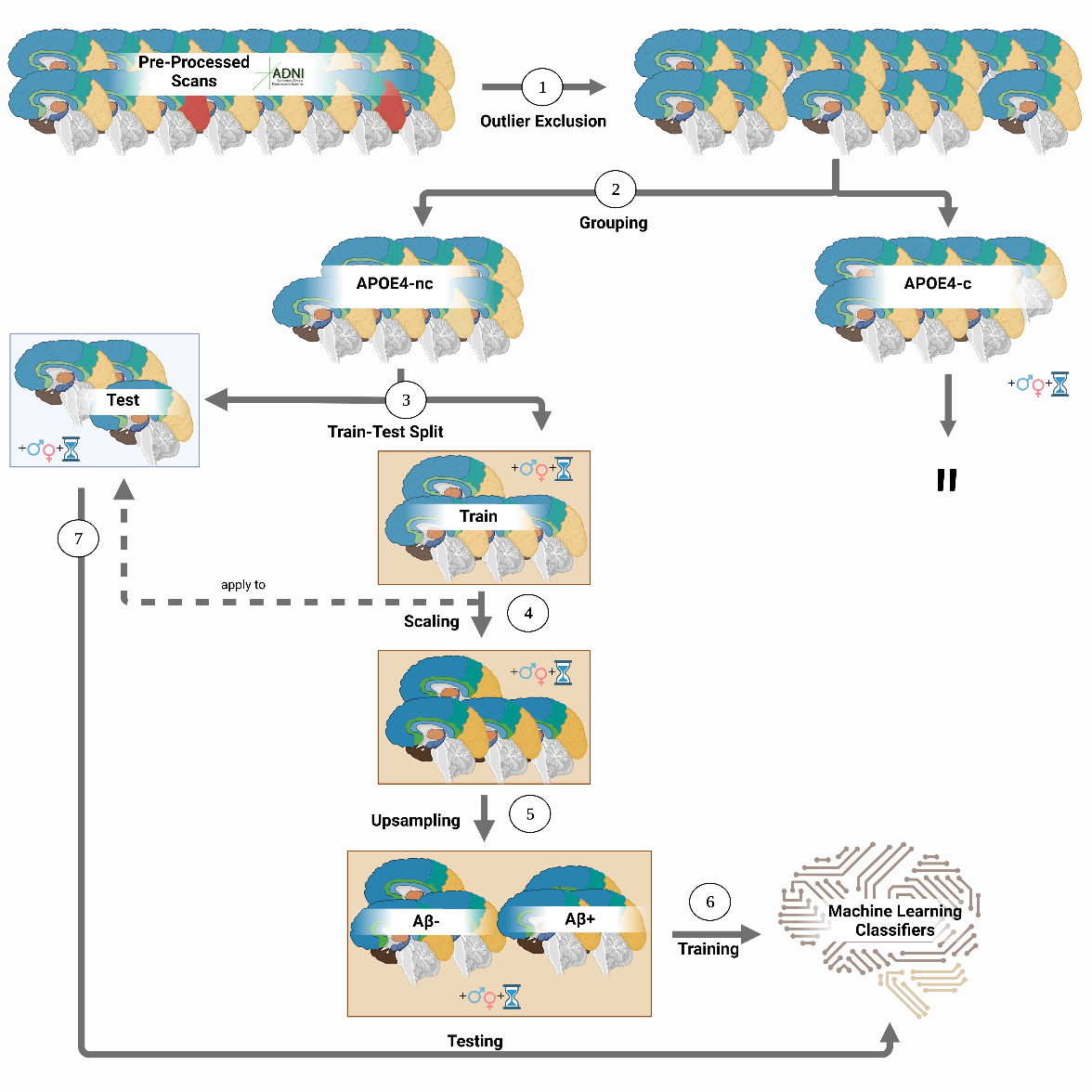


Fig. S1 Pipeline of the gatekeeping system. After exclusion of MCI patients whose ^18^F-FDG-PET scan showed mean regional SUVRs outside three times the interquartile range (1), individuals were split into groups of APOE4-nc and APOE4-c (2). After grouping, the pipeline followed a similar standard procedure in both groups. For visibility, only APOE4-nc classification from FDG-PET (brains), age (hourglass) and sex (gender signs) is depicted here. (3) Groups were split into 70% train and 30% test data. (4) Scaling transformations were estimated on the train set and applied to both train and test set. (5) The train set was upsampled so that numbers of Aβ- and Aβ+ were equalized. (6) Classifiers were trained using the train set. (7) Classifiers were tested on the ADNI and IMC test set.


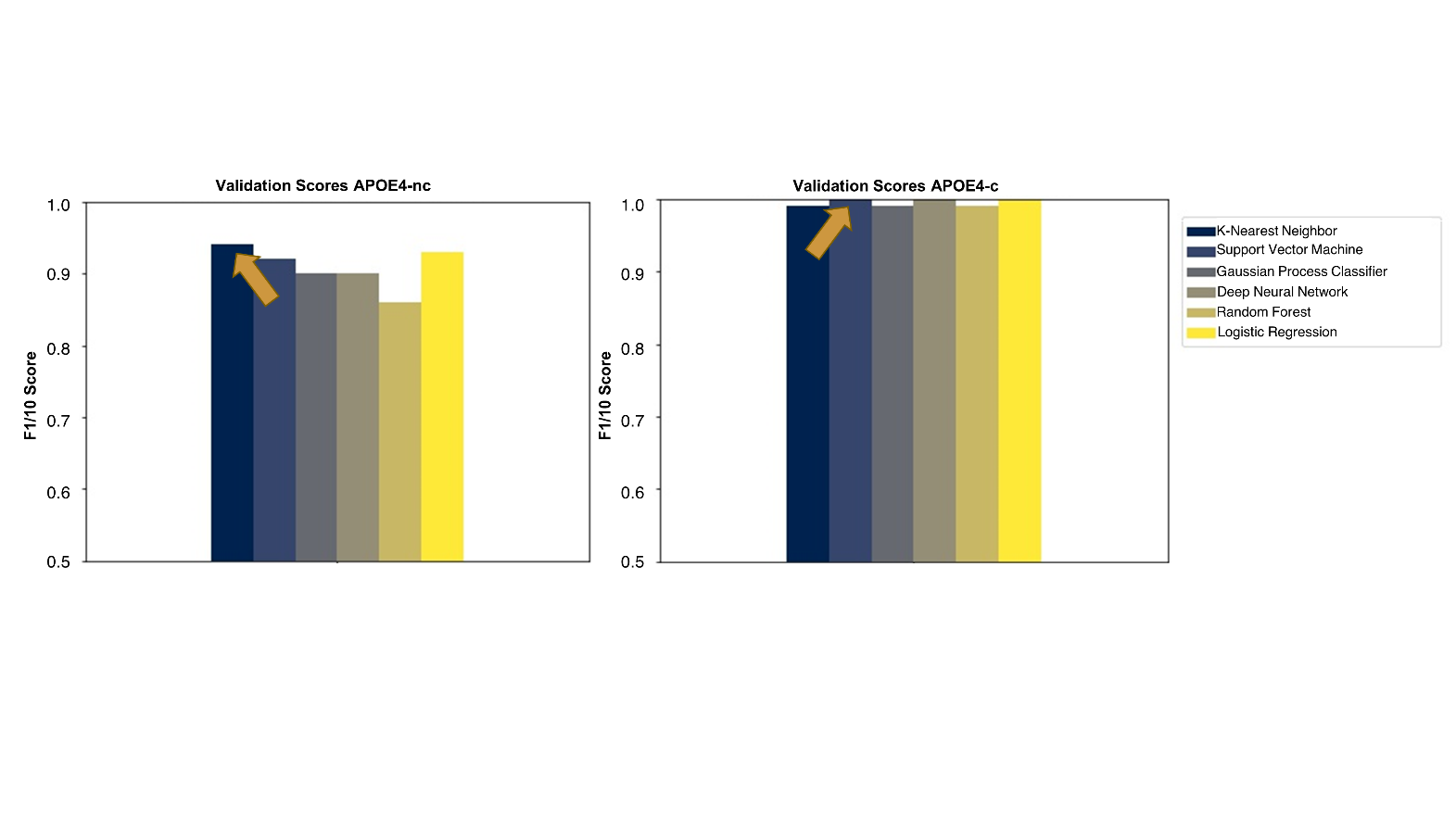


Fig. S2 Classification performance of intermediate models during cross-validation in the APOE4-nc (left) and APOE4-c group (right). Arrows point to the choice of final models
